# Efficient Integrity-Tree Structure for Convolutional Neural Networks through Frequent Counter Overflow Prevention in Secure Memories

**DOI:** 10.3390/s22228762

**Published:** 2022-11-13

**Authors:** Jesung Kim, Wonyoung Lee, Jeongkyu Hong, Soontae Kim

**Affiliations:** 1 School of Computing, Korea Advanced Institute of Science and Technology (KAIST), 291, Daehak-ro, Yuseong-gu, Daejeon 34141, Republic of Korea; 2 Department of Computer Engineering, Yeungnam University, 280 Daehak-Ro, Gyeongsan, 38541, Gyeongbuk, Republic of Korea

**Keywords:** hardware security, secure memory, integrity-tree, counter overflow, convolutional neural network

## Abstract

Advancements in convolutional neural network (CNN) have resulted in remarkable success in various computing fields. However, the need to protect data against external security attacks has become increasingly important because inference process in CNNs exploit sensitive data. Secure Memory is a hardware-based protection technique that can protect the sensitive data of CNNs. However, naively applying secure memory to a CNN application causes significant performance and energy overhead. Furthermore, ensuring secure memory becomes more difficult in environments that require area efficiency and low-power execution, such as the Internet of Things (IoT). In this paper, we investigated memory access patterns for CNN workloads and analyzed their effects on secure memory performance. According to our observations, most CNN workloads intensively write to narrow memory regions, which can cause a considerable number of counter overflows. On average, 87.6% of total writes occur in 6.8% of the allocated memory space; in the extreme case, 93.9% of total writes occur in 1.4% of the allocated memory space. Based on our observations, we propose an efficient integrity-tree structure called Countermark-tree that is suitable for CNN workloads. The proposed technique reduces overall energy consumption by 48%, shows a performance improvement of 11.2% compared to VAULT-128, and requires a similar integrity-tree size to VAULT-64, a state-of-the-art technique.

## 1. Introduction

Machine learning (ML) technologies based on convolutional neural networks (CNN) have matured over the past several years and are actively employed in real-life applications such as personal voice assistants, smart home systems, autonomous driving cars, and medical diagnosis [1,2,3]. However, providing sufficient security remains an open problem. Recent studies underline the possible threats to CNN-based applications, such as deceiving self-driving cars to classify road signs incorrectly [4] and injecting adversarial data resulting in an inaccurate medical diagnosis [5]. In [6,7,8], service providers are cautioned about direct damage occurring owing to stolen internal neural network information, such as network topologies and learned weight values that are priceless intellectual properties. Because such information is usually retained in the main memory, a high security level of the main memory through secure memory is required to protect the assets of the neural network. However, it is difficult to apply secure memory in an Internet of Things (IoT) environment that requires space efficiency and low power consumption.

In order to ensure the security of the main memory, integrity and confidentiality must be satisfied [9,10,11,12]. Confidentiality is achieved by encrypting data going from the processor to the external untrusted area. Integrity ensures that the main memory correctly returns the last written data block to any address. An *integrity tree* is used to satisfy integrity and prevent replay attacks [9,10,11,12]. In counter-mode encryption, counters are managed by an integrity tree, where a memory write increases the corresponding counter. In case of a counter overflow, every correlated memory block is re-encrypted [13,14,15]. There are many techniques for preventing frequent counter overflows, as discussed in [9,13,14,15,16]. In particular, in [13], the authors proposed a variable *n*-arity tree structure with counters of different sizes for different tree levels. In general applications, it is difficult to generalize memory access patterns and predict overheads in secure memory. However, CNN workloads exhibit distinctive memory access patterns regardless of the CNN model type. These patterns cause significant performance degradation in secure memory and render the previously proposed secure memory techniques ineffective. Therefore, it is necessary to understand the memory access patterns of CNN workloads in order to efficiently use secure memory.

In this paper, we investigated the memory access patterns for CNN inferences and analyzed their effects on the performance of secure memory. In our study, most CNN inference workloads intensively write to narrow memory regions owing to of the characteristics of the CNN topologies. In addition, the output size was reduced across the layers. A CNN consists of several layers, where the output of one layer is used as the input for the next layer. These outputs are repeatedly stored in the same physical memory buffer, resulting in numerous memory writes to narrow memory regions. On an average, 87.6% of total writes occurred in 6.8% of the allocated memory space. In the extreme case, 93.9% of total writes occurred in 1.4% of the allocated memory space. This characteristic can cause high overhead owing to frequent counter overflows in secure memory. Therefore, a technique that can suppress counter overflows while maintaining area efficiency is required for CNNs.

Based on these observations, we propose *Countermark-tree*, an efficient integrity-tree structure suitable for CNN inference workloads. The Countermark-tree allocates different counter sizes within the same tree level depending on the write intensity of the corresponding memory block. Because CNN inference workloads write intensively only in a small memory region and rarely write to the remaining area, almost no overflow occurs for most counters. On average, counters corresponding to only 6.8% of the total memory used caused overflows. This implies that counter size should be adjusted within the same integrity-tree level to reduce counter overflows for CNN workloads. Our proposed scheme is based on the internal characteristics of the programming model, not the structure of the CNN itself or the structures of accelerators. To the best of our knowledge, this is the first proposed CNN programming model-based secure memory technology.

We evaluated Countermark-tree with CNN inference workloads and compared our design to a baseline using VAULT-64 and VAULT-128, which contain 64 and 128 counters, respectively, per counter node. Our scheme shows an 11.2% performance improvement over VAULT-128, and it requires less than half the space required by VAULT-64 without additional performance overhead. In summary, this paper makes the following contributions:

1. **CNN feature analysis for secure memory**: We analyze the impact of CNN workloads on secure memory and the inefficiency of previously proposed techniques.
2. **Countermark-tree**: We propose an integrity-tree design for CNN workloads that reduces overflow by assigning counters of different sizes depending on the memory block write intensities.

The rest of this paper is organized as follows. In Section 3, we explain the background and motivation behind this study. Section 4 introduces the proposed scheme and Section 5 presents the experimental results. Section 2 summarizes previous studies in the literature. Finally, Section 6 concludes this paper.

## 2. Related Works

**Integrity-tree for secure memory:** Previously proposed techniques mitigate overheads in an integrity-tree via caching. To overcome the coverage limitations of the integrity-tree, the tree node can be modified such that a memory block can contain more metadata. For this purpose, tamper-evident counter tree (TEC-tree) [17], parallelizable authentication tree [18], and SGX tree [19] have been proposed. These methods use a counter-tree-based integrity-tree, where the arity of the tree depends on the number of counters in a node. For further improvements, the split counter scheme [14] divides a counter into a large major counter and small minor counters in a memory block to minimize the counter overflow overhead. In other studies, techniques for secure memory using non-volatile memory have been proposed [20,21,22,23] to solve endurance and consistency problems cause by secure memory. A recent work [13] entailed the development of an integrity-tree structure called VAULT having different characteristics by exploiting the fact that higher-level counters increase faster than lower-level counters.

**Neural network security:** Tramèr et al. [6] proposed a model extraction attack aimed at duplicating the functionality of CNN models in an ML-as-a-service environment. Yan et al. [7] applied the previously suggested cache attack schemes to steal CNN models. Hua et al. [8] suggested a possible attack technique that uses an Intel SGX-like system. They pointed out that Intel SGX provides an isolated and protected execution environment; however, memory inputs and outputs remain exposed to an adversary. These studies imply that there are vulnerabilities in modern CNN environments that should be addressed in the literature. In [24,25], the authors proposed a secure execution environment for GPUs. Because these techniques are not limited to neural networks, they do not utilize related properties. NPUFORT [26] proposes a customized accelerator that encrypts security-critical features. TNPU [27] focuses on providing custom accelerators with security guarantees provided by traditional security processors, such as Intel SGX. Although various studies have been conducted before, there is no secure improvement technique based on the programming model characteristics of ML applications.

## 3. Background and Motivation

In this section, we provide a brief introduction to secure memory primitives, including trusted computing base and convolutional neural network, respectively.

### 3.1. Security Primitives

#### 3.1.1. Trusted Computing Base

A modern secure system model assumes that on-chip processor components, such as cores, registers, and caches, are protected from physical attacks. External memory and peripherals are considered vulnerable components. Based on this assumption, the following security concepts are considered to provide safe and secure environments:

- **Confidentiality**: To prevent an attacker from reading the memory content, the data transmitted outside of the secure processor are encrypted. Every encryption operation is performed using a unique key.
- **Integrity**: Although an attacker cannot violate confidentiality, they can violate integrity by injecting arbitrary data into a requested address (*spoofing*), assigning a value to another address (*splicing*), and reusing old data to a previously available address (*replay*).

#### 3.1.2. Counters for Data Encryption

**Figure 1:**
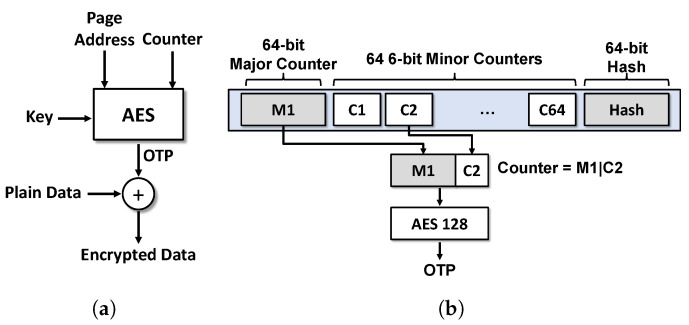
(**a**) Encryption module used in counter-mode encryption and (**b**) a tree node of a split counter [14].

In secure memory, counter-mode AES encryption [9,13,28], which can encrypt/decrypt memory blocks in parallel, is mainly used to reduce extra read latency. In counter-mode encryption, the key used for data encryption should not be used more than once (key reuse problem [9,14,19,29]). A one-time pad (OTP) is generated by the encryption data address and counter with an encryption key. Because the OTP bits have strong entropy, a sufficient security level of the data can be guaranteed by XORing with the OTP to the data bits. To ensure the uniqueness of the encryption keys, a counter value is used with the encryption key for data encryption, as shown in Figure 1a. This encryption approach is called *counter-mode encryption* [14], in which counters exist for each memory block. Every memory write increases the corresponding counter; therefore, the counter can overflow owing to frequent writes. During an overflow, every counter is reset, and the corresponding memory blocks are re-encrypted, which causes significant additional memory traffic. Therefore, the counter bits should be sufficiently long to avoid frequent counter overflow. Note that counters for data are called *encryption counters*, and counters for an encryption counter are called *integrity-tree counters*.

#### 3.1.3. Split Counter Scheme

A counter structure called a *split counter* [14] has been proposed to maximize the coverage of secure memory with a reduced counter size. In a split counter, 64 or 128 minor counters and one major counter are included in one 64-byte memory block, and each minor counter corresponds to a 64-byte memory block. These counter blocks are organized in tree form, which is called an integrity-tree. As shown in Figure 1b, the counter is divided into a single large major counter and several small minor counters, and each minor counter is associated with a memory block of data. These two types of counters are used as the prefix and postfix, respectively, and are used after concatenation when used as an encryption key. When a counter overflow occurs in a minor counter, the major counter is incremented by one, and all minor counters in the node are reset to 0. In addition, the corresponding memory blocks are re-encrypted. This structure is advantageous for extending the arity of an integrity-tree by packing a number of logical counters in a node. For this purpose, the split counter scheme can be applied to store 64 6-bit counters in a node (defined as a *split counter-64*) which covers 64 memory blocks of data. Furthermore, it can be configured to pack more counters into a node that has 128 3-bit minor counters, which is defined herein as a *split counter-128*.

#### 3.1.4. Replay-Attack Protection with Integrity-Tree

**Figure 2:**
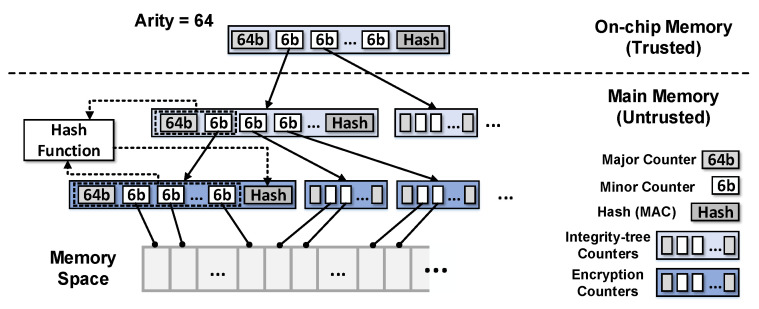
Overall structure of the counter-tree.

Even if the attacker cannot violate confidentiality through encryption, they can violate integrity by spoofing, splicing, and replay attacks. Among these attacks, spoofing and splicing can be prevented via data and message authentication code (*MAC*) pair comparison; however an integrity-tree [19,29] should be used to protect against replay attacks. The integrity-tree consists of multiple MAC levels, with each level ensuring the integrity of the lower level. In addition, the root of the integrity-tree is stored in a secure processor to ensure integrity. In counter-mode encryption, the integrity of the counters is guaranteed by a counter-tree [19,29]. The counter-tree uses a hashing algorithm to generate MACs using an integrity-tree counter. As shown in Figure 2, an MAC is generated using the counters present in the node and a counter of the corresponding parent node to ensure the integrity of a counter-tree node. In general, the size of a counter-tree node is the same as that of a memory block (64-byte). Therefore, it is important to strike a balance between the number of counters and bit length of each counter to minimize the occurrence of counter overflow and maximize the coverage of secure memory.

#### 3.1.5. VAULT

**Figure 3:**
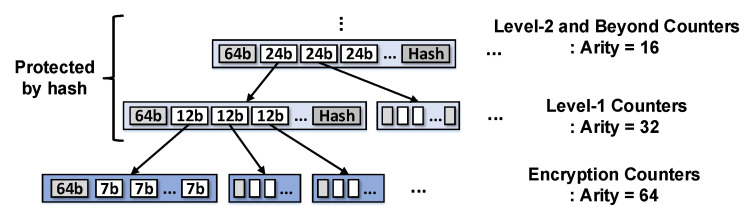
The tree structure of VAULT [13].

In [13], a variable-arity unified encrypted leaf tree (VAULT) was demonstrated. In the counter-tree-based integrity-tree, the counters at higher levels are saturated faster than those at lower levels because the increment of the counter value that occurs at the leaf node propagates to the root; hence, overflows to the higher-level counters occur frequently. To address this problem, the tree structure of VAULT is designed to have more bits in the counter as the node at a higher level, as shown in Figure 3. This variable-arity design permits less overflow, even for high traffic conditions occurring at higher levels of the tree.

Similar to the split-counter scheme, VAULT can have 64 counters in a node (*VAULT-64*). In contrast with the split-counter scheme, VAULT does not store a hash in the leaf node. Therefore, the minor counter of the VAULT-64 encryption counters has one more bit (7-bit) than that of the split-counter scheme; accordingly, it can reduce the overflow probability by half. We extend VAULT to store 128 minor counters for more aggressive counter packing (*VAULT-128*). Finally, we compared our proposed technique with VAULT, the latest scheme for integrity-tree structure.

### 3.2. Neural Network

#### 3.2.1. CNN Structure and Inference

**Figure 4:**
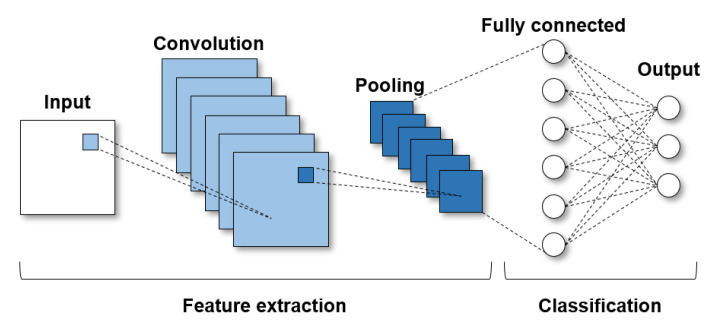
Basic CNN architecture.

A CNN consists of multiple layers, where each layer can be defined by a filter and activation function. Figure 4 represents the basic CNN architecture. In feature extraction, there is a convolution process that separates and identifies various features of an image for analysis. Several pairs of convolution and pooling layers constitute a feature extraction network. After feature extraction, a fully connected layer utilizes the output of the convolution process and predicts the class of input images. To forward the input data to the next layer, a convolution is applied to the input data with the filter. The result of the convolution is passed through the activation function, and the result, called the *feature map*, becomes the input for the next layer. The CNN inference uses only this simple forwarding procedure, whereas the training includes highly computation-intensive backward propagation. Therefore, CNNs can be operated in relatively lightweight environments, such as automobiles, smartphones, and IoT devices [30].

#### 3.2.2. Forwarding Operation and Memory Access

**Figure 5:**
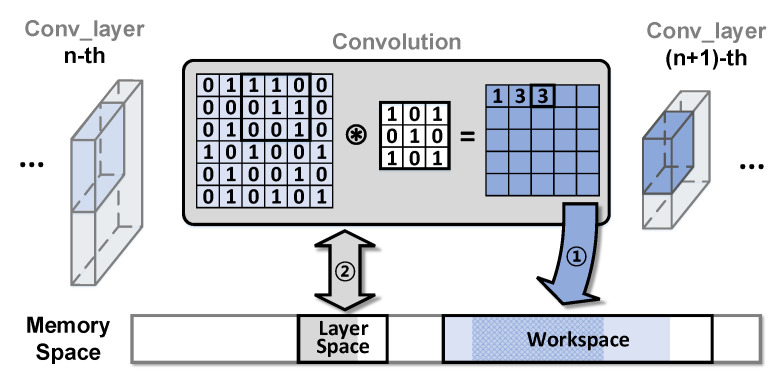
Forwarding procedure and its memory access pattern.

Figure 5 depicts the forwarding procedure and memory access patterns. The forwarding operation during inference can be divided into three steps. First, the input feature maps and filter weights are imported from the memory. The filter weights are stored in the independent memory area for each layer, called the *layer space*, and feature maps are stored in the shared memory area among the layers, called the *workspace*. This is a reasonable implementation in terms of memory efficiency, as the filter weights should be maintained by each layer; however, the input feature maps are no longer required after the forwarding operation. Second, convolution and activation are performed based on the loaded feature maps and filter weights. Typically, the size of the feature maps is reduced after convolution. During these procedures, the intermediate calculation results should be maintained in the layer space. The last step stores the output feature maps in to the workspace area. The previous feature maps are no longer used, and the old feature maps are overwritten by the new output feature maps.

#### 3.2.3. Problematic Overwrites on Narrow Memory Space

Intensive writes on hotspots cause severe performance degradation in secure memory. During the forwarding operation, there is chance that performance degradation may occur. First, overwriting in the workspace can cause degradation (see arrow ① in Figure 5). The workspace is repeatedly overwritten to store the newly generated feature maps at the end of each forwarding operation. Although the size of overwritten space reduces gradually, the hotspot remains until the end of the inference process. Second, the intermediate results of the convolution can cause performance degradation (see arrow ② in Figure 5). If the convolution calculation is too memory-intensive this can produce a large amount of data, resulting in many overwrites in the layer space. In Section 3.3, we demonstrate the effects of intensive overwrites on performance degradation.

### 3.3. Motivation: Memory Access Behavior of CNNs

In general, CNN models require frequent memory access to load and store feature maps as well to maintain intermediate calculation results. In order to mitigate the performance and energy overhead caused by these characteristics, previous studies [31] have analyzed the memory access pattern and proposed a dedicated accelerator or programming model using GPGPU. However, running a CNN on secure memory requires additional performance overhead to satisfy security requirements.

As mentioned in Section 3.1, modern secure memory implementations use counter-mode encryption to ensure data confidentiality and integrity. Each time a memory block is written to the main memory, the corresponding counter value is increased. When this counter value is saturated, counter overflow occurs, which results in memory re-encryption. In the split-counter scheme [14], every minor counter included in one counter node must be read and re-encrypted, i.e., 64 counters for split counter-64 and 128 counters for split counter-128. Therefore, as counter overflow occurs more frequently, performance becomes severely degraded. Overflow occurs more frequently when the workload is memory write-intensive and a small portion of memory is repeatedly written.

**Figure 6:**
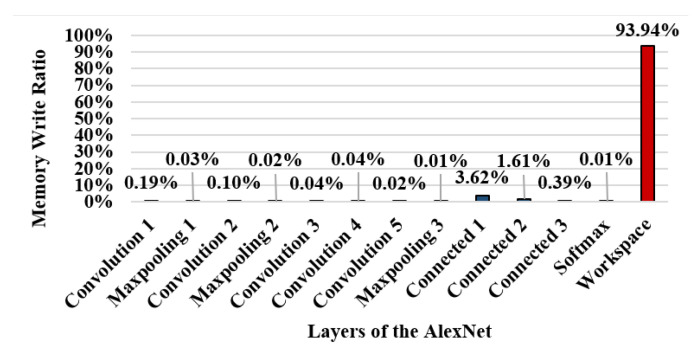
Memory write ratio per layer in AlexNet.

Figure 6 shows the write ratio in the memory area of each layer during a single inference operation of AlexNet [32]. The X-axis represents the inner layers of AlexNet, as described in Figure 4. Note that the workspace is not a logical layer of AlexNet; rather, it is a buffer memory area allocated to share feature maps between layers. The percentage of writes in the workspace area is 93.94% of the total writes, indicating that most writes occur in the workspace.

**Figure 7:**
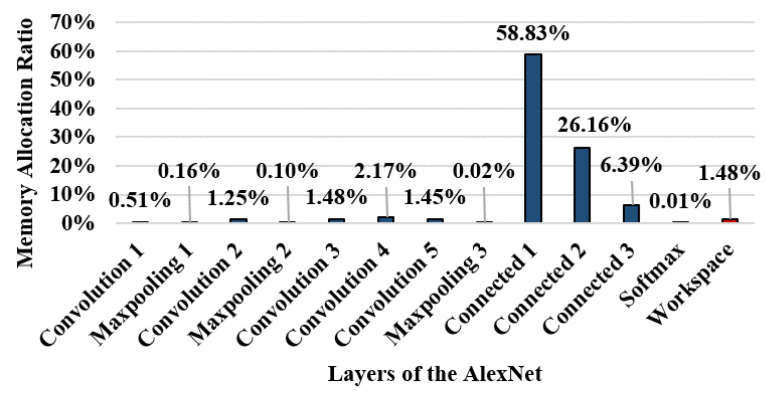
Allocated memory ratio in AlexNet.

Figure 7 shows the ratio of the memory areas used by each layer of AlexNet. The workspace memory size is only 1.48% of the total memory used by AlexNet; however 93.94% of the write operations occur in this area. Thus, AlexNet has a strong write hotspot, which causes many writes to occur in the narrow memory area, resulting in numerous re-encryptions when running on secure memory.

Table 1 shows the percentage of write hotspots where more than 80% of the total memory writes occured for the twelve CNN models: AlexNet, Darknet [33], Extraction [33], ResNet [34], ResNext [35], and VGG-16 [36]. Apart from VGG-16, an average of 6.8% of memory blocks generate 80% of the total memory writes in the CNN models. Because the size of the feature maps is relatively large in VGG-16, a larger capacity is required to store the intermediate calculation results. Overall, strong hotpots tend to form irrespective of the CNN model type, filter type, and size.

## 4. Countermark-Tree

In this section, we present our proposed technique, the Countermark-tree. We demonstrate the inefficiency of previously proposed schemes in CNN applications, then provide a design methodology for our proposed method.

### 4.1. Inefficiency of Previously Proposed Schemes

Because of the inherent characteristics of CNN inference workloads, which are distinctly different from the workload of general applications, previously proposed secure memory techniques are not effective for CNNs. Table 2 shows the number of re-encryptions per million instructions that occur when the twelve CNN models perform inference on the secure memory. As mentioned in Section 3.1.5, the encryption counter of VAULT-64 uses one bit more than does that of split counter-64. Thus, on average, VAULT-64 has fewer re-encryption frequencies than split counter-64. In the case of VAULT-128, the additional bit is not available in the encryption counter. Therefore, the counter overflow frequency is not much different from that of split counter-128; there is only a small benefit from the VAULT structure, because the overflow in the encryption counter is dominant among the total overflows occurring in the entire integrity-tree.

**Figure 8:**
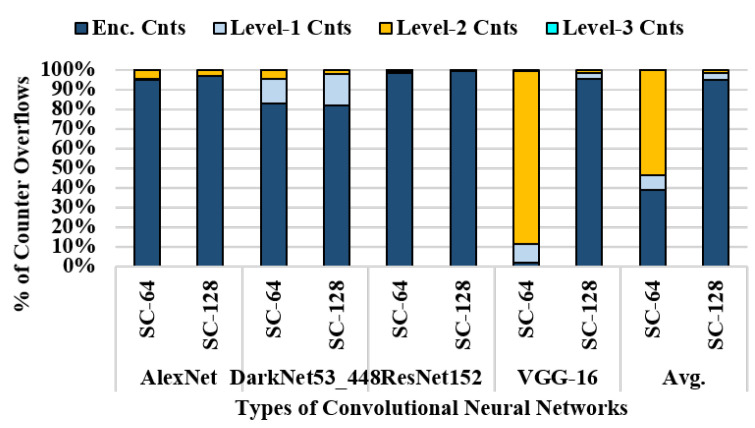
Counter overflow breakdown for split counter-64 and split counter-128.

Figure 8 shows the breakdown of the total counter overflow regarding four different CNN workloads. Regardless of the counter size, the overflows occurring in the encryption counters are dominant, except for VGG-16. The encryption counter is incremented when the corresponding memory block is evicted from the last-level cache (LLC). Similarly, integrity-tree counters are incremented when a child counter is evicted from the metadata cache. Because the data reuse distance for most CNN workloads can be covered in the LLC, only a small number of encryption counters are accessed, and the metadata cache is efficiently utilized. Evictions in the metadata cache rarely occur, and more than 80% of overflows are from the encryption counter. However, owing to the large working set of VGG-16, frequent evictions occur in the LLC and metadata cache in the case of split counter-64. As a result, the metadata cache miss rate increases, and numerous overflows arise in integrity-tree counters.

**Figure 9:**
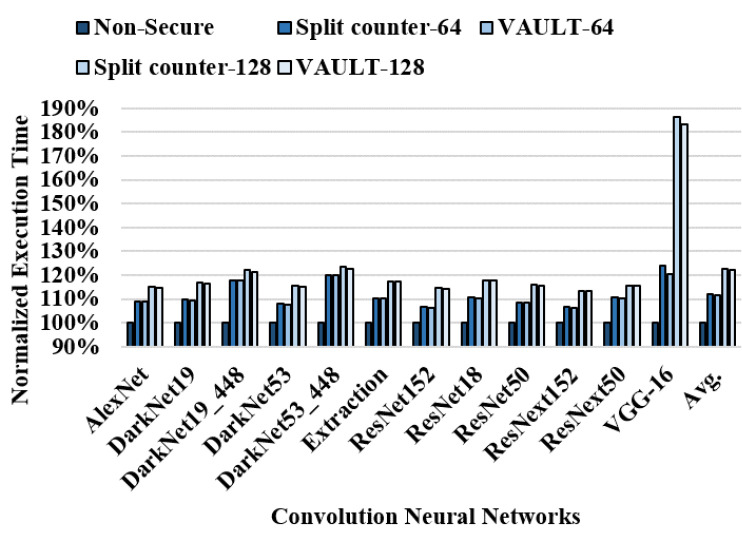
Normalized execution time for split counter-64, VAULT-64, split counter-128, and VAULT-128.

Figure 9 shows the normalized execution time for CNN workloads. An average slowdown of 11.8%, 11.4%, 22.8%, and 22.4% occurred in the split counter-64, VAULT-64, split counter-128, and VAULT-128, respectively. As discussed in Section 3.3, numerous writes occur in a narrow memory region and almost no writes occur in most other regions. The frequent overflows in the narrow memory region cause significant performance overhead, which makes it impossible to reduce the overall counter size. Therefore, in order to effectively suppress the counter overflow caused by the CNN workload it is necessary to allocate counters of different sizes according to the memory area write intensity.

### 4.2. Designing Countermark-Tree

As mentioned in Section 3.3, in a CNN it is ineffective to differentiate the arity according to the level of the integrity-tree such as VAULT, as shown in the case of VAULT- 128. Therefore, it is necessary to adjust the arity horizontally rather than vertically.

**Figure 10:**
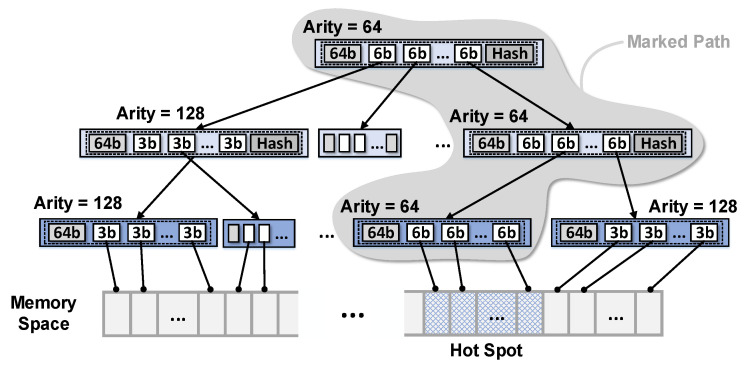
The structure of Countermark-tree.

Figure 10 shows the structure of the proposed Countermark-tree scheme, which allocates more counter bits to the hotspot memory region. For general applications, the size and location of memory hotspots vary depending on the type of application, values of inputs, and walking set size. Therefore, it is difficult to predict which parts of memory become hotspots and how large they might be. However, CNN inference always performs the same process regardless of the input value, and its memory access pattern is very predictable as well. Therefore, memory hotspot locations can be predicted in advance, and the frequency of overflow can be suppressed by allocating more bits to the counter associated with the hotspot. The paths from the hotspot to the root are defined as *marked paths*, and the counters included in the marked path are defined as *marked counters*. The marked counters should have more bits to suppress the overflows triggered by the hotspot; accordingly, each counter extends its counter bits from 3-bit to 6-bit. This *marked counter extension* technique ensures that the counters are at least eight times more robust against the overflows on the hotspot. As mentioned in Section 3.3, CNN workloads intensively write to a small area, and rarely write to other areas. These features provide the opportunity to work efficiently when larger counters are allocated along a marked path. In addition, the storage overhead can be minimized by maintaining the counter bits in normal areas.

### 4.3. Implementation Methodology

We assume that software supports secure memory, similar to Intel SGX (Software Guard eXtension) [37], which is the latest industrial implementation of secure memory. In SGX, sensitive code and data are stored in a module called *enclave*. If the SGX user-runtime triggers enclave access, the hardware SGX module checks the access permission and address range in the privileged environment.

In a CNN, the hotspot area is determined by the CNN topology and algorithm regardless of other parameters, such as input data and weight values. Therefore, it is possible to statically analyze and determine the address range of the hotspot before runtime. Subsequently, information about the address range of the hotspot can be delivered by the memory controller register, which in SGX is the processor reserved memory region register (PRMRR) [37]. The number of registers required depends on the CNN topology. Table 3 shows the size of the hotspots, the number of writes covered by the hotspot, and the number of chunks that represent the number of consecutive allocated memory areas for each CNN workload. As shown in the table, hotspots comprise a small amount of consecutively allocated memory in most cases, except for ResNext152 and ResNext50. Contrary to the common case, in ResNext152 and ResNet50 it is possible to group several chunks together because of the short distance between them. Therefore, only a small number of registers are required to store the address range of the hotspots. In the case of VGG-16, for example, while the hotspot size is 480 MB, the entire region is continuously allocated to four chunks. Consequently, it is possible to determine whether a write request is heading towards the hotspot by comparing the start and end addresses of the hotspot stored in the registers. Note that our proposed scheme cannot be dynamically applied to all models of CNN at runtime. Memory information about the CNN to be used must be delivered to the memory controller in advance before the CNN application is performed.

## 5. Evaluation

In this section, we perform an in-depth study on our proposed scheme. The experimental configurations listed in Table 4 are used to reveal its performance and energy consumption.

### 5.1. Experimental Methodology

#### 5.1.1. Simulation Configuration

We used the USIMM [38] simulator as the main experimental framework. Table 4 shows the system configuration. The last-level cache size is reduced from 4MB to 1MB, as CNN inference can be performed with other general applications. Based on the baseline system, we implement a dual-AES module for data encryption consisting of a Bonsai Merkle tree [29] and split-counter [14]. We used the Synergy [15] design to eliminate MAC overheads. We implemented the VAULT [13] architecture as a state-of-the-art technique for comparison with the our proposed scheme. This experimental environment was operated by running memory traces, and the Sniper [39] simulator and Darknet [33] CNN framework were used to generate memory traces.

#### 5.1.2. CNN Workloads

We evaluated the proposed scheme using twelve CNN workloads [32,34,35,36] taken from the Darknet [33] framework. Table 5 lists the number of memory accesses per kilo instructions (MPKI) and size of the memory footprints for each CNN workload. Each number after the CNN name represents the depth of the respective CNN model; a deeper model requires more memory space, and thus has larger memory footprints. The 448 in Darknet represents the input image size, which is larger than the normal size (=256); therefore, there are relatively more read operations in this framework than those in the normal Darknet models for reading larger images.

### 5.2. Impact on Storage Overhead

In Countermark-tree, the 64-ary counter is applied to the memory blocks of the write hotspot and the 128-ary counter is used for the others in order to maximize space efficiency and minimize overflow. Table 6 shows the storage overhead of the split counter, VAULT, and Countermark-tree schemes. All the schemes reported in Table 6 cover the same size of secure memory. Unlike other schemes, in Countermark-tree (CM-tree in the table), the number of bits used in the encryption counter or integrity-tree counter is determined depending on the size of the hotspot formed owing to each CNN workload. For example, in the case of VGG-16, which has the largest hotspot area, the hotspot size is 480MB, meaning that 128.1MBis allocated for the encryption counters and 1.11MB for the integrity-tree counters. Compared with VAULT-64, the proposed scheme requires only 48.76% of the memory space needed for VGG-16, and requires 48.8% on average.

### 5.3. Impact on Performance

**Figure 11:**
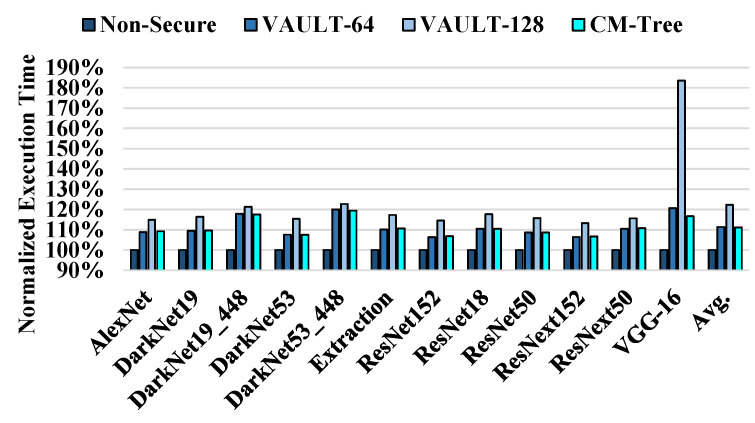
Normalized execution time for VAULT-64, VAULT-128, and Countermark-tree.

Figure 11 shows a normalized performance comparison between VAULT-64, VAULT-128, Countermark-tree, and the non-secure baseline. Countermark-tree shows a 0.3% and 11.2% speed increase compared with VAULT-64 and VAULT-128, respectively. Therefore, compared with VAULT-64, our scheme can achieve almost the same performance with only half the space overhead. This performance benefit results from the reduced miss rate of the metadata cache, as the number of counters that can be loaded into a single 64-byte cache line increases. The innate characteristics of the CNN inference workload form the hotspots, and the metadata on hotspot blocks are frequently accessed. Therefore, the metadata cache is highly utilized, resulting in a low miss rate. In the case of VGG-16, the metadata cache miss rates are 17.5%, 3.7%, and 3.4% for VAULT-64, VAULT-128, and Countermark-tree, respectively. The performance gain of Countermark-tree in VGG-16 is larger than that in the other CNN models because the miss rate compared to VAULT-64 is reduced by 14%.

### 5.4. Analyzing Extra Memory Traffic

**Figure 12:**
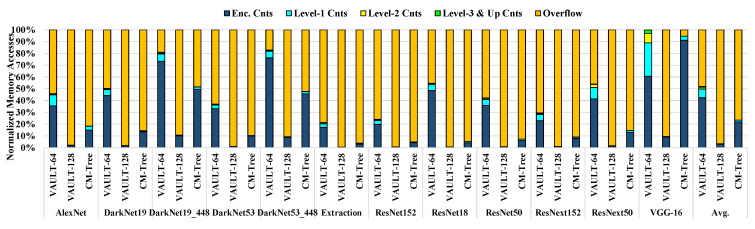
Extra memory traffic for VAULT-64, VAULT-128, and Countermark-tree.

Figure 12 shows the additional memory traffic for VAULT-64, VAULT-128, and Countermark-tree. For VAULT-64 and VAULT-128, the percentages of additional memory traffic owing to overflows account for more than 48% and 96%, respectively. These results point to a different traffic aspect compared to the perspective of previous studies [13], as a CNN has different characteristics from general applications. VAULT is not suitable for CNN applications, as discussed in Section 3.2.3, because intensive writes are performed in a narrow memory space. In the case of VAULT-64, over 50% of the extra traffic is caused by the overflow; accordingly, overflows mostly happen in the encryption counters. Although VAULT-64 has one more bit in the encryption counter, the counter is quickly saturated because the update frequency of the hotspot is too high. Furthermore, VAULT-64 has the highest integrity-tree level, which causes more conflict misses in the metadata cache than other techniques. Consequently, more misses of the integrity-tree counters occur, resulting in frequent memory accesses to fetch these occurrences. In the case of VAULT-128, although more bits are allocated for the higher-level counters, it cannot leverage VAULT’s structure because of frequent overflows in the encryption counter.

Table 7 represents the number of overflows per million instructions in VAULT-64, VAULT-128, and Countermark-tree. For most CNN applications, VAULT-128 suffers from severe counter overflows against VAULT-64. In particular, a CNN with a large number of layers, such as VGG-16, shows a significant increase in overflows. However, our Countermark-tree shows that overflows can be suppressed sufficiently even in the case of VGG-16. This indicates that our proposed technique can suppress counter overflows by allocating more counter bits to the memory area where writes are performed intensively, thereby minimizing performance degradation. On average, 2.1, 48.9, and 2.4 overflows per million instructions occur in VAULT-64, VAULT-128, and Countermark-tree, respectively. Although the counter size of Countermark-tree is one bit smaller than that of VAULT-64, most counters are 128-ary; aaccordingly, more counters can be loaded in the metadata cache. Therefore, fewer metadata cache misses occur, and overall memory accesses are reduced.

### 5.5. Impact on Power and Energy Consumption

**Figure 13:**
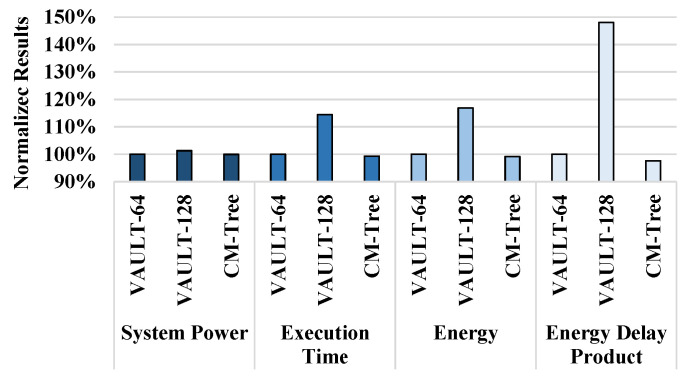
Power, execution time, energy, and EDP for VAULT-64, VAULT-128, and Countermark-tree.

Figure 13 shows the system power, execution time, energy, and energy-delay product (EDP) for VAULT-64, VAULT-128, and Countermark-tree. Note that system power represents the power consumed by the processor. Countermark-tree has virtually the same the counter structure compared to that of the split-counter and VAULT schemes. Therefore, there is little difference between these three methods in terms of system power consumption, as they do not participate in the operation of the processor. Frequent counter overflows caused by VAULT-128 cause the memory controller to undergo more stall cycles. Therefore, VAULT-128 requires more execution time. However, our proposed scheme shows a similar level of execution time compared to VAULT-64. Note that additional memory traffic due to counter overflows does not directly affect performance. If normal memory operation requested from the processor is delayed due to additional traffic, this causes performance degradation. However, if additional traffic occurs when the memory is idle, it does not directly cause performance degradation. As shown in Figure 11, the execution time of the proposed scheme (0.7% faster) is similar to that of VAULT-64. This is because the performance loss from the frequent overflows in Countermark-tree offsets the performance gain from the reduction in metadata cache misses. The energy consumption of Countermark-tree is approximately 0.9% less than that of VAULT-64. The EDP of VAULT-128 is 48% higher than that of VAULT-64, and the EDP of Countermark-tree is approximately 2.4% lower than that of VAULT-64.

## 6. Conclusions

CNNs can be executed in secure memory to protect their data, including sensitive input data and learned weight values, severe performance degradation is caused owing to characteristics of CNN workloads. In this paper, we investigated the memory access patterns for various CNN models and analyzed their effect on performance. CNNs have a strong hotspot, which causes many writes in a narrow memory space and reduces the effectiveness of previously proposed secure memory techniques. Based on these observations, we propose Countermark-tree, which is a simpler and more efficient integrity-tree structure than existing schemes. The proposed scheme performs comparably to VAULT-64, while the area overhead is reduced by half. The performance and energy consumption overhead are reduced by 11.2% and 48%, respectively, compared to those of VAULT-128, while requiring similar area overhead.

## Figures and Tables

**Table 1:** Percentage of memory region that covers more than 80% of total writes.

Workloads	Normal	Hotspot
AlexNet	0.9864	0.0136
DarkNet19	0.9357	0.0643
DarkNet19_448	0.9946	0.0054
DarkNet53	0.9683	0.0317
DarkNet53_448	0.9137	0.0863
Extraction	0.9712	0.0288
ResNet152	0.9908	0.0092
ResNet18	0.9405	0.0595
ResNet50	0.9650	0.0350
ResNext152	0.9699	0.0301
ResNext50	0.9596	0.0404
VGG-16	0.5921	0.4079
Avg.	0.9323	0.0677

**Table 2:** Number of overflows per million instructions for split counter-64, VAULT-64, split counter-128, and VAULT-128.

Workloads	Split Counter-64	VAULT-64	Split Counter-128	VAULT-128
AlexNet	4.02	1.72	21.11	20.40
DarkNet19	6.41	2.86	33.07	31.65
DarkNet19_448	3.14	1.29	18.56	15.27
DarkNet53	7.30	3.41	38.60	37.46
DarkNet53_448	2.80	1.18	15.85	13.22
Extraction	5.61	2.71	29.77	29.58
ResNet152	6.11	2.90	32.81	32.64
ResNet18	6.82	3.12	34.22	33.88
ResNet50	5.69	2.52	30.40	30.11
ResNext152	4.52	2.18	24.78	24.55
ResNext50	2.24	1.01	13.79	13.60
VGG-16	34.21	0.36	317.52	304.18
Avg.	7.40	2.10	50.87	48.88

**Table 3:** Hotspot size and number of memory chunks.

Workloads	Alloc. Hotspot	Write Intensity	# of Chunks
AlexNet	6.7 MB	94.39%	1
DarkNet19	18 MB	96.37%	1
DarkNet19_448	55.1 MB	81.71%	1
DarkNet53	18 MB	96.54%	1
DarkNet53_448	93.4 MB	80.11%	7
Extraction	7 MB	97.85%	1
ResNet152	9.2 MB	83.74%	1
ResNet18	9.2 MB	98.17%	1
ResNet50	15.2 MB	81.02%	3
ResNext152	41.8 MB	80.18%	38
ResNext50	20 MB	80.02%	10
VGG-16	480 MB	80.95%	4

**Table 4:** Experimental system configuration.

Configuration	Value
Number of cores Processor clock speed Processor ROB size Processor fetch/retire width	4 3.2 GHz 192 4
Last level cache (Shared) Metadata cache (Shared)	1 MB *, 8-way, 64 B lines 128 KB, 8-way, 64 B lines
Memory size Memory bus speed Bank, Rank, Channels Rows per bank Cache lines per row	16 GB 800 MHz 8, 2, 2 64 K 128
Page allocation policy	Random
Encryption latency	40 ns

* Reduced size for access behavior isolation of single workload.

**Table 5:** Memory access per kilo instructions and memory footprint.

Workloads	Read-PKI	Write-PKI	Footprint (GB)
AlexNet	17.9	16.7	0.3
DarkNet19	33.7	24.2	0.2
DarkNet19_448	37.4	11.8	0.4
DarkNet53	34.9	28.5	0.4
DarkNet53_448	39.5	10.2	0.8
Extraction	35.6	22.6	0.2
ResNet152	27.0	24.8	0.7
ResNet18	39.3	26.2	0.1
ResNet50	28.8	22.9	0.3
ResNext50	16.4	9.6	0.4
ResNext152	22.1	19.5	1.0
VGG-16	37.3	7.8	1.1

**Table 6:** Storage overheads for 16 GB memory.

Configuration	Tree Depth	Enc. Cnts	Integrity-Tree
split counter-64	3	256 MB	4.06 MB
VAULT-64	6	256 MB	8.59 MB
split counter-128	2	128 MB	1.01 MB
VAULT-128	3	128 MB	2.06 MB
CM-tree(VGG-16)	2	128.1 MB	1.11 MB
CM-tree(Avg.)	2	128 MB	1.01 MB

**Table 7:** Overflows per million instructions for VAULT-64, VAULT-128, and Countermark-tree.

Workloads	VAULT-64	VAULT-128	CM-Tree
AlexNet	1.72	20.40	2.00
DarkNet19	2.86	31.65	3.14
DarkNet19_448	1.29	15.27	1.69
DarkNet53	3.41	37.46	3.56
DarkNet53_448	1.18	13.22	1.47
Extraction	2.71	29.58	2.82
ResNet152	2.90	32.64	3.07
ResNet18	3.12	33.88	3.20
ResNet50	2.52	30.11	2.84
ResNext152	2.18	24.55	2.24
ResNext50	1.01	13.60	1.16
VGG-16	0.36	304.18	1.68
Avg.	2.10	48.88	2.41

## Data Availability

Not applicable.
